# Mechanisms of Oxidative Stress in Metabolic Syndrome

**DOI:** 10.3390/ijms24097898

**Published:** 2023-04-26

**Authors:** Sepiso K. Masenga, Lombe S. Kabwe, Martin Chakulya, Annet Kirabo

**Affiliations:** 1 HAND Research Group, School of Medicine and Health Sciences, Mulungushi University, Livingstone Campus, Livingstone P.O. Box 60009, Zambia; 2 Department of Medicine, Room 536 Robinson Research Building, Vanderbilt University Medical Centre, Nashville, TN 37232-6602, USA

**Keywords:** oxidative stress, inflammatory cytokines, metabolic syndrome, obesity, hypertension, insulin resistance, hyperglycemia, hyperlipidemia, cardiovascular disease

## Abstract

Metabolic syndrome is a cluster of conditions associated with the risk of diabetes mellitus type 2 and cardiovascular diseases (CVDs). Metabolic syndrome is closely related to obesity. Increased adiposity promotes inflammation and oxidative stress, which are precursors of various complications involving metabolic syndrome components, namely insulin resistance, hypertension, and hyperlipidemia. An increasing number of studies confirm the importance of oxidative stress and chronic inflammation in the etiology of metabolic syndrome. However, few studies have reviewed the mechanisms underlying the role of oxidative stress in contributing to metabolic syndrome. In this review, we highlight mechanisms by which reactive oxygen species (ROS) increase mitochondrial dysfunction, protein damage, lipid peroxidation, and impair antioxidant function in metabolic syndrome. Biomarkers of oxidative stress can be used in disease diagnosis and evaluation of severity.

## 1. Introduction

Metabolic syndrome is characterized by the presence of several interconnected risk factors for type 2 diabetes (T2DM) and cardiovascular disease (CVD) [1]. The presence of metabolic syndrome increases the risk of developing T2DM by 5 fold, CVD by 2 fold, and the risk of all-cause mortality by 1.5 fold [2,3]. Metabolic syndrome is highly prevalent in the United States, with about 35% prevalence and almost half in those aged 65 years and older [2,4]. The risk factors of metabolic syndrome include increased waist circumference or belly fat, high plasma triglycerides, elevated blood pressure, high blood sugar, and low plasma high density lipoprotein (HDL) [1]. If a patient has three of the five major risk factors, a diagnosis of metabolic syndrome is made [5].

Although many factors contribute to the pathophysiology of metabolic syndrome, several studies show that oxidative stress, in conjunction with chronic inflammatory conditions, is at the core of the development of metabolic diseases [4,6,7]. The imbalance between oxidants and antioxidants, which is often tilted in favor of the oxidants, is what causes oxidative stress, which in turn causes a disruption in redox signaling and regulation as well as molecular and cellular damage [8,9]. Metabolic syndrome is characterized by obesity-related problems, indicating a relationship between obesity and metabolic syndrome [10]. Inflammation and oxidative stress play a significant role in the development of metabolic comorbidities such as hyperlipidemia, high blood pressure, and increased glucose intolerance, all of which lead to metabolic dysfunction [10,11]. Several studies have shown that the risk for metabolic syndrome can be greatly reversed by reducing body weight and focusing interventions on dietary changes such as time-restricted eating, special diets such as the Mediterranean diet, including increasing physical exercise, sleep changes, or even reduce stress [2,3,5,12]. In this review we explore the mechanisms involving oxidative stress in metabolic syndrome. We discuss mechanisms associated with each component of metabolic syndrome and a few related risk factors associated with metabolic syndrome.

## 2. Metabolic Syndrome Components

Metabolic syndrome is characterized by abdominal obesity, high blood pressure, insulin resistance (a risk factor for T2DM), a proinflammatory and prothrombotic state, and atherogenic dyslipidemia (high triglycerides, high apolipoprotein B, high low-density lipoprotein particle (LDL-p) number, and low high-density lipoprotein cholesterol (HDL-C) [3,5,13,14]. The components of metabolic syndrome are interrelated, as briefly described below. Obesity is the primary risk factor in the development of T2DM, and it is estimated that roughly 90 percent of people who have T2DM are either overweight or obese [15,16]. Obesity is also linked to an increased risk of CVD, which includes conditions such as high blood pressure, atherosclerosis, acute myocardial infarction, and heart failure [4]. Central obesity is defined as having an abdominal circumference that is greater than 102 cm for males and greater than 88 cm for women [17]. Central obesity is one of the most important factors in the etiology of metabolic syndrome, including insulin resistance [18]. Obesity is associated with low-grade inflammation, which may lead to insulin resistance, insulin deficiency, and metabolic disturbances [4]. Insulin, a peptide hormone released by pancreatic beta cells in response to rising blood glucose, blocks lipolysis, and hepatic gluconeogenesis, and increased glucose absorption in the liver, muscles, and adipose tissues [19]. Insulin resistance is an altered physiologic response to insulin stimulation of the target tissues, such as the liver, muscle, and adipose tissue, and the resistance hinders glucose metabolism, resulting in hypertrophy of beta cells, increased beta-cell insulin production, and hyperinsulinemia [20,21]. Insulin resistance may result in hyperglycemia, hypertension, dyslipidemia, visceral obesity, hyperuricemia, increased inflammatory markers, endothelial dysfunction, and thrombosis, which may lead to metabolic syndrome, nonalcoholic fatty liver disease (NAFLD), and T2DM through several complex mechanisms [21,22,23].

Lipid metabolism is critical to the etiology of insulin resistance and the subsequent development of metabolic syndrome [24]. Lipid changes also contribute to the diagnostic criteria for metabolic syndrome, and the two major lipids include fasting triglyceridemia >150 mg/dL and HDL cholesterol concentration <40 mg/dL. This lipid derangement is characterized by an increase in the synthesis of very low-density lipoproteins (VLDL), a decrease in the plasma’s lipolytic capacity, and an increased cholesterol ester transfer protein activity [18]. Patients with metabolic syndrome also exhibit hemostatic changes that can elevate the risk of both atherothrombotic and thromboembolic cardiovascular disease [18,25]. The atherothrombotic and thromboembolic changes result from endothelial dysfunction, which may be caused by chronic inflammation, dyslipidemia, and hypertension [18,25].

## 3. Mechanisms of Reactive Oxygen Species and Their Role in the Development and Progression of Metabolic Syndrome

**Figure 1:**
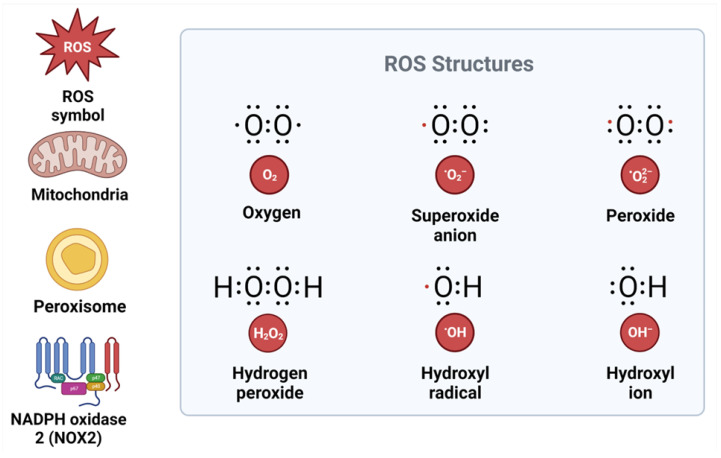
Structure of reactive oxygen species and their sources. ROS, reactive oxygen species; NADPH, nicotinamide adenine dinucleotide phosphate; NOX2, NADPH oxidase.

ROS production is tightly regulated by redox signaling and sensing mechanisms [26] (Figure 1).

**Figure 2:**
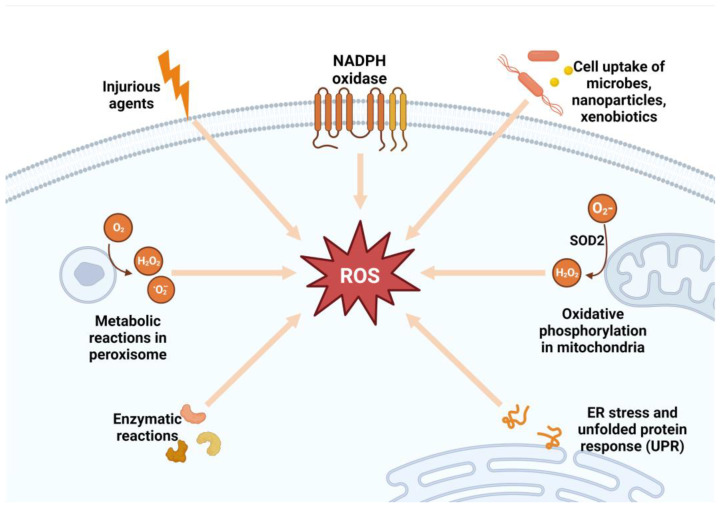
Sources of reactive oxygen species. SOD2, superoxide dismutase 2; ROS, reactive oxygen species; ER, endoplasmic reticulum; NADPH, nicotinamide adenine dinucleotide phosphate; H_2_O_2_, hydrogen peroxide.

ROS signaling may participate in normal physiological processes or contribute to maladaptive responses that result in metabolic dysfunction and inflammatory signaling, depending on the ROS source, cell type, and tissue environment [9,27,28]. The two major sources of ROS inside the cell are nicotinamide adenine dinucleotide phosphate (NADPH) oxidase (NOX) enzymes and the mitochondria [29]. The NOX enzymes are a family of enzymes (NOX1, NOX2, NOX3, NOX4, NOX5, DUOX1, and DUOX2) located in the cell membrane, and NOX2-NOX3 is important in most pathological conditions [29]. In the mitochondria, ROS are formed during oxidative phosphorylation by oxidizing reduced nicotinamide adenine dinucleotide (NADH) to NAD^+^ [30,31]. The superoxide anion that is produced by the mitochondria and NOX2 is rapidly converted by an enzyme called superoxide dismutase into hydrogen peroxide (H_2_O_2_), which serves as a signaling molecule [32,33] (Figure 2). Hydrogen peroxide is a powerful oxidizing agent. For this reason, cells express antioxidant proteins, including peroxiredoxin, catalase, glutathione (GSH), and thioredoxin, that convert H_2_O_2_ to water [31,34]. The level of H_2_O_2_ must be strictly maintained; hence, its production must be equal to its reduction [9]. High H_2_O_2_ in the presence of free ferric iron (Fe^2+^) produces hydroxyl radicals (•OH) in the Fenton reaction [9,28].

Through tightly controlled redox regulation, signaling, and sensing, ROS are essential for normal biological functions in physiologic settings [35]. Oxidative posttranslational modification (Ox-PTM), also known as oxidative protein modification, is a crucial molecular process that regulates proteins, which eventually affect the biological responses of cells [36]. Redox-sensitive proteins include ion transporters, receptors, signaling molecules, transcription factors, cytoskeletal structural proteins, and matrix metalloproteases [9]. Proteins are normally targets of reversible Ox-PTM; however, in pathological conditions associated with oxidative stress, such as hypertension, proteins undergo irreversible Ox-PTM, which results in a loss of protein function and, as a consequence, cell damage, tissue injury, and failure of the target organs [37,38]. ROS, such as H_2_O_2,_ are also essential for the activation of cellular pathways, including those that interact with vasoactive drugs such as angiotensin II (Ang II), endothelin-1 (ET-1), aldosterone, and prostanoids used to mediate cellular effects, and those that regulate intracellular calcium homeostasis [9]. ROS activate transcription factors such as hypoxia-inducible factor (HIF) that regulates angiogenesis, activate the phosphoinositide 3 kinase (PI3K) pathway that regulates cellular growth, the nuclear factor kappa-light-chain-enhancer of activated B cells (NF-kB) pathway, which in normal conditions prevents apoptosis by regulating cell survival, activates the mitogen-activated protein kinase (MAPK) pathway, which regulates cellular proliferation [39]. ROS also stimulate the transcription of pro-inflammatory chemokine and cytokine production and the recruitment and activation of inflammatory and immune cells [40,41].

**Figure 3:**
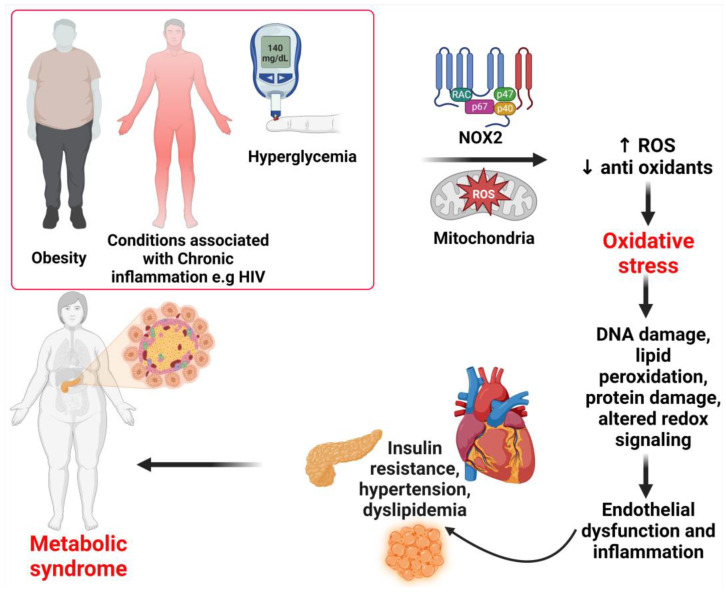
Mechanisms of metabolic syndrome. Under pathological conditions such as obesity, chronic inflammation, and hyperglycemia, excessive ROS generation can occur. ROS production occurs through the activation of enzymes in the cytosol, membrane, and mitochondria. An increase in the production of ROS and the depletion of antioxidants result in oxidative stress. The resulting oxidative stress leads to intracellular cell damage and altered redox, which leads to the irreversible accumulation of oxidation products, promoting endothelial dysfunction, which leads to insulin resistance, hypertension, dyslipidemia, and, subsequently, metabolic syndrome. ROS, reactive oxygen species; NOX2, nicotinamide adenine dinucleotide phosphate (NADPH) oxidase (NOX) enzymes.

Overproduction of ROS can occur in pathological disorders such as obesity, insulin resistance, hyperglycemia, chronic inflammation, and dyslipidemia [40,42,43]. Oxidative stress is detrimental because all the excess ROS induces cellular damage, specifically damaging DNA and peroxidize lipids [44]. Lipids present in plasma, mitochondrial, and endoplasmic reticulum membranes are major targets of ROS attack and peroxidation in most macromolecules [30,44]. The end products of lipid peroxidation, known as lipid peroxides, can be toxic to a cell and require removal by glutathione through an elusive mechanism [45]. Many studies have found that metabolic syndrome patients had lower plasma antioxidant enzyme activity and greater biomarkers of oxidative damage than healthy individuals, which may contribute to oxidative stress [46]. In the same manner, proteins and nucleic acids can be subject to peroxidation as well as nitrosylation [31]. Nevertheless, these end products are not usually directly toxic to the cell [31]. However, accumulation of inactive proteins can overload the ability of a cell to metabolize them and hence lead to the damage of DNA as they are capable of activating apoptosis [45]. In addition, the accumulation of modified proteins decreases their function, leading to a severe loss of normal cell activity [4,27,32,45]. The overproduction of ROS results in an oxidative stress environment, which also destabilizes redox signaling and control and leads to deleterious effects on gene expression, increases growth factors and stress response elements, and activates the apoptosis pathway [9,27]. The disrupted redox signaling also promotes pro-inflammatory and pro-fibrotic pathways, which affect insulin metabolic signaling and endothelial dysfunction and promote cardiovascular and renal inflammation and fibrosis, which contribute to target organ damage [9,47]. The mechanisms of ROS and their role in the development of metabolic syndrome are shown in Figure 3.

## 4. Mechanisms of Oxidative Stress Associated with Abdominal Obesity

### 4.1. Inflammation and Free Radical Production via Several Pathways

Obesity is a metabolic disorder characterized by either an excessive accumulation of body fat (BF) or an improper distribution of BF that is associated with adverse effects [32,48,49]. Obesity can be both a result of and a cause of oxidative stress [50]. Excessive intake of lipids, carbohydrates, and saturated fatty acids, particularly trans-fatty acids, stimulates specific intracellular pathways, leading to oxidative stress through superoxide generation via oxidative phosphorylation, glyceraldehyde autoxidation, protein kinase C activation, and activation of the polyol and hexosamine pathways [43,50,51]. Animal and cell culture studies have shown that oxidative stress may play a causative role in obesity by increasing pre-adipocyte proliferation, differentiation, size, white adipose tissue (WAT), and alter food intake [43,50].

Obesity can lead to systemic oxidative stress due to increased NOX activity and ER stress in adipocytes, as well as abnormal post-prandial metabolism, ROS generation, hyperleptinemia, chronic inflammation, tissue dysfunction, and low antioxidant defenses [50,51,52]. Oxidative stress and inflammation are closely linked to obesity. In adipocytes of obese individuals, there is activation of pro-inflammatory transcription factors, such as NF-κB and activator protein-1 (AP-1), which are redox-sensitive and trigger the release of inflammatory cytokines such as tumor necrosis factor alpha (TNF-α), interleukin-1β (IL-1β) and interleukin-6 (IL-6), which in turn enhance ROS production, creating a vicious circle [43,53,54]. Oxidative stress and inflammation are important components in the pathophysiology of obesity-related conditions such as atherosclerosis, insulin resistance, type 2 diabetes, and cancer [55].

**Figure 4:**
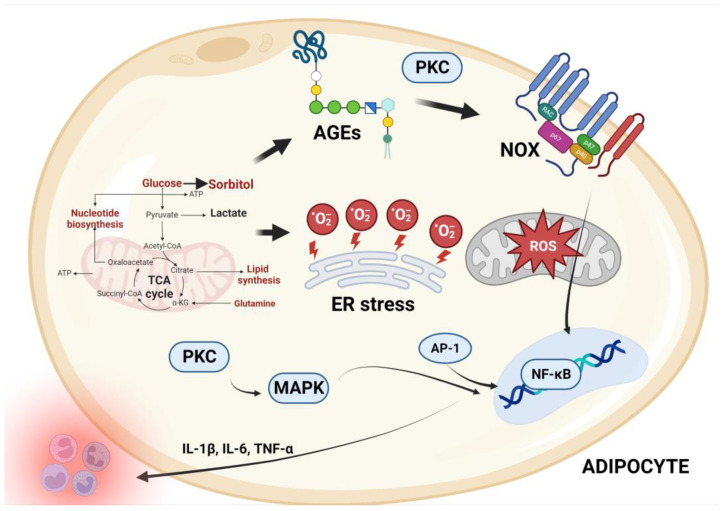
Proposed mechanisms of oxidative stress associated with adipocytes. Nutritional excess and adipocyte hypertrophy, as well as the release and accumulation of pro-inflammatory mediators such as free fatty acids (FFA), hyperglycemia, advanced glycation end products, cytokines, and pro-inflammatory cytokines linked to protein kinase C (PKC) and polyol pathways, characterize obesity. By activating NADPH oxidase (NOXs), nitric oxide synthase, uncoupled endothelial NOS (eNOS), and myeloperoxidase, these components may induce tissue oxidative stress. Chronic inflammation may also contribute to the modification of adipose tissue’s redox balance by activating stress signal transduction, which contributes to increased autophagy and apoptosis, uncontrolled adipokine production, and adipose tissue inflammation. The resultant functional changes may further impair adipose tissue function by affecting intracellular pathways that generate pro-inflammatory cytokines, resulting in increased attraction, infiltration, and activation of immune cells, as well as increased adipose tissue inflammation, thereby creating a vicious cycle between adipose tissue oxidative stress and inflammation, as well as a decrease in antioxidant system activity, ultimately leading to metabolic dysfunction. AGEs, advanced glycation end products; PKC, protein Kinase C; NOX, nicotinamide adenine dinucleotide phosphate oxidase enzyme; ER, endoplasmic reticulum; MAPK, mitogen-activated protein kinase; NF-kB, nuclear factor kappa-light-chain-enhancer of activated B cells; ROS, reactive oxygen species; TCA, tricarboxylic cycle; TNF-α, tumor necrosis factor alpha.

Numerous mechanisms, including altered lipid and glucose metabolism (hyperglycemia), chronic inflammation, tissue dysfunction, hyperleptinemia, and aberrant post-prandial ROS formation, have been proposed to increase oxidative stress in obese people [43,51,56]. Glycolysis and the tricarboxylic acid (TCA) cycle produce the electron donor’s nicotinamide adenine dinucleotide hydrogen (NADH) and reduced flavin adenine dinucleotide (FADH_2_) [57,58]. In overnutrition, excessive glucose increases metabolism via glycolysis and the TCA cycle, resulting in increased NADH and FADH_2_ formation in the mitochondrial electron transport chain [50,51,56]. The increased proton gradient causes electron leakage and causes reactive intermediates to produce superoxide anions in addition to those produced by activated NADPH oxidase [50,51,56]. Through the enzyme superoxide dismutase, superoxide is converted to hydrogen peroxide [59]. The free radical inhibits glyceraldehyde-3-phosphate dehydrogenase and consequently shifts upstream metabolites into four alternate pathways, which increase free radical generation or reduce antioxidant defenses, causing oxidative/nitrosative stress [50]. The four alternative pathways include the following: (1) Activation of the polyol pathway, which involves the reduction of glucose into sorbitol via aldolase reductase, which uses NADPH, resulting in depletion of cytosolic NADPH and subsequently increased ROS production [60,61,62]. (2) Fructose-6-phosphate is converted to glucosamine-6-phosphate, which inhibits thioredoxin action and causes oxidative and ER stress [50]. (3) Triose phosphates produce methylglyoxal, the main precursor of advanced glycation end products (AGEs) [63]. AGEs activate NOX pathways, which increase the production of ROS/reactive nitrogen species (RNS), whereas NF-κB alters gene expression and causes transcription of pro-inflammatory cytokines (including TNF-α and IL-6), adhesion molecules, microRNAs (miR), and inducible nitric oxide synthase (iNOS), which are implicated in adipogenesis, inflammation, and oxidative stress [47,63,64]. (4) Dihydroxyacetone phosphate is converted to diacylglycerol, which activates the protein kinase C (PKC) pathway, which plays a vital role in the development of cardiovascular complications via its activation of MAPK cascades (Figure 4) [51,55,65].

Obesity is linked to an increase in plasma free fatty acids (FFA) and excessive fat storage in white adipose tissue (WAT) [66,67]. The pathological increase in serum FFA levels caused by excessive fat accumulation in obese people impedes glucose metabolism, enhances hepatic, muscle, and adipose accumulation of energy substrates, and increases mitochondrial and peroxisomal oxidation [50,55]. Adipose tissue is a major source of ROS production as it promotes the generation of superoxide ions in the mitochondrial electron transport chain by inhibiting adenine nucleotide translocation [56], leading to oxidative stress, mitochondrial DNA damage, ATP depletion, and lipotoxicity. This causes an increase in the production of cytokines such as TNF-α, which in turn generates more ROS in the tissues and worsens lipid peroxidation [55].

The proinflammatory cytokines TNF-α, IL-1, and IL-6 have been linked to adiposity [68]. TNF-α regulates the inflammatory response, immune system, adipose cell apoptosis, lipid metabolism, hepatic lipogenesis, insulin signaling, and oxidative stress [43,49,69]. Obesity increases serum TNF-α, which induces the release of IL-6 from immune cells and adipocytes and reduces systemic anti-inflammatory cytokines, promoting systemic inflammation [50,70]. Tissue dysfunction amplifies oxidative stress and inflammation, leading to increased expression of adipokines, deletion of nuclear factor E2-related factor 2 (Nrf2), and endothelial dysfunction in obesity and obesity-induced hypertension [55,71]. Angiotensin II (Ang II) regulates IL-6 and TNF-α secretion, allowing monocyte recruitment and exacerbating vascular injury [55,72]. Monocytes emit the pyrogenic cytokine IL-1β after tissue injury, infection, or immunologic insult [73]. Production of pro-inflammatory cytokines, including IL-1β and IL-6, has been linked to obesity’s pro-inflammatory response [55,72]. IL-6 regulates energy homeostasis and inflammation, affecting the transition from acute to chronic inflammatory diseases, such as obesity and insulin resistance, through promoting the synthesis of pro-inflammatory cytokines and negatively regulating inflammatory targets [72,74]. Higher serum IL-6 levels are associated with decreased glucose tolerance, diabetes mellitus, high blood pressure, and obesity in humans [72,74].

### 4.2. Adipokines

Bioactive adipokines such as leptin, adiponectin, visfatin, resistin, apelin, and plasminogen activator inhibitor type 1 (PAI-1) are found in adipose tissue and have been linked to the homeostasis of physiological and pathological processes involving oxidative stress [75,76]. Adipocytes secrete leptin in proportion to adipose tissue mass and triglyceride accumulation [77]. Leptin promotes hunger through its action in the central nervous system (CNS) [55]. Hyperleptinemia increases oxidative stress and stimulates the proliferation and activation of monocytes/macrophages, producing IL-6 and TNF-α [51]. Leptin also activates NOX and induces the production of reactive intermediates such as H_2_O_2_ and OH free radicals [50]. It also decreases the activity of the cellular antioxidant paraoxonase-1 (PON-1), a decrease that is associated with increased levels of plasma and urinary F(2)-isoprostane (8-isoPGF2), and plasma levels of malondialdehyde and hydroperoxides [56,78,79]. Adiponectin is important in glucose and lipid metabolism and helps to avoid the development of pathological changes [80]. Adiponectin works as an anti-inflammatory and anti-atherogenic hormone secreted by differentiated adipocytes, which decreases TNF-α and C-reactive protein (CRP) levels, increases NO production, and inhibits ROS release [81,82]. Its serum levels are inversely correlated with systemic oxidative stress [81,82]. Visfatin is a pleiotropic molecule showing pro-oxidant and pro-inflammatory effects, and its levels are positively correlated with body fat mass, and its concentration decreases when weight loss occurs [81].

### 4.3. Food Intake

The post-prandial response to high-fat and high-carbohydrate (HFHC) meals is impaired in obese people, which could lead to an increase in oxidative stress [51]. Obese individuals exhibit a more pronounced and prolonged oxidative and inflammatory response to HFHC meals, as well as increased expression of the p47phox subunit of NOX2, increased ROS generation, intra-nuclear NF-кB binding in mononuclear cells, and plasma matrix metalloproteinase (MMP-9) concentrations [54,83].

Vitamin and mineral deficiencies can also contribute to the development of compromised antioxidant defense in the pathophysiology of obesity [50]. Obese people are more susceptible to oxidative damage due to decreased antioxidant sources and significantly decreased antioxidant activity [50]. Antioxidant supplementation reduces oxidative stress and ROS, lowers obesity-related comorbidities, and restores adipokine expression [55,84].

## 5. Mechanisms of Oxidative Stress Associated with Abnormal Lipogram Levels

Lipoproteins are complex molecules that have a central hydrophobic core of non-polar lipids, primarily cholesterol esters and triglycerides [85]. The non-polar lipid is engulfed by a hydrophilic membrane consisting of phospholipids, free cholesterol, and apolipoproteins [86]. Frequently, most of the lipids that fall under the category of lipoproteins and estimated lipid markers include total cholesterol, high-density lipoprotein cholesterol (HDL-C), low-density lipoprotein cholesterol (LDL-C), and triglycerides [31,86]. One of the mechanisms through which the accumulation of lipids begins to form a plug in the blood vessels is linked to excessive biosynthesis of ROS, which leads to oxidative stress in the walls of the blood vessels [85]. An increase in the magnitude of ROS supports the oxidation process of LDL. This results in a high level of oxidized LDL (ox-LDL), thereby resulting in the death of vascular endothelial cells, and subsequent endothelial dysfunction [86]. Furthermore, the oxidation of LDL leads to further oxidation in the vascular walls hence resulting in increased levels of lipid hydroperoxides such as the lipid hydroperoxides (LOOH) in the LDL [87]. At the cellular level, particularly in mitochondria, there is dysregulation of oxidative metabolism, resulting in unbalanced levels of ROS biosynthesis [44,88]. The imbalance leads to disrupted mitochondrial utilization of lipids, resulting in accumulation in body tissues [44,88]. At the micro level, ROS disrupt cell signaling and cause mitochondrial dysfunction, resulting in an energy deficit and ultimately, function loss [44]. It is imperative to note that the citric acid cycle intermediate molecule, citrate, is transported to the cytoplasm, where it is used as a substrate in the production of fatty acids (FA) and cholesterol. However, when the mitochondria are damaged due to ROS accumulation, HDL and cholesterol metabolism will be defective, suggesting that ROS contribute to the elevation of both molecules [31].

## 6. Mechanisms of Oxidative Stress Associated with Hypertension

Hypertension is one of the most significant cardiovascular risk factors for metabolic syndrome [7,89]. Many factors contribute to the development of hypertension, as proposed by Irvine Page, when he developed the mosaic theory of hypertension, which states that multiple factors, including genetics, environmental factors, adaptive and endocrine factors, and hemodynamic forces, all contribute to the development of hypertension [90,91]. Since then, great strides have been made to explain the molecular and cellular basis of hypertension, including the discovery of nitric oxide (NO) and its role in the cardiovascular system and the role of oxidative stress in factors associated with the mosaic theory [91,92]. Current evidence indicates that oxidative stress is a significant contributor to the development of hypertension. Oxidative stress and chronic inflammation have been linked to endothelial damage and vascular dysfunction, cardiovascular remodeling, renal dysfunction, sympathetic nervous system excitation, immune cell activation, and systemic inflammation that lead to high blood pressure and heart diseases [7,93,94,95].

**Figure 5:**
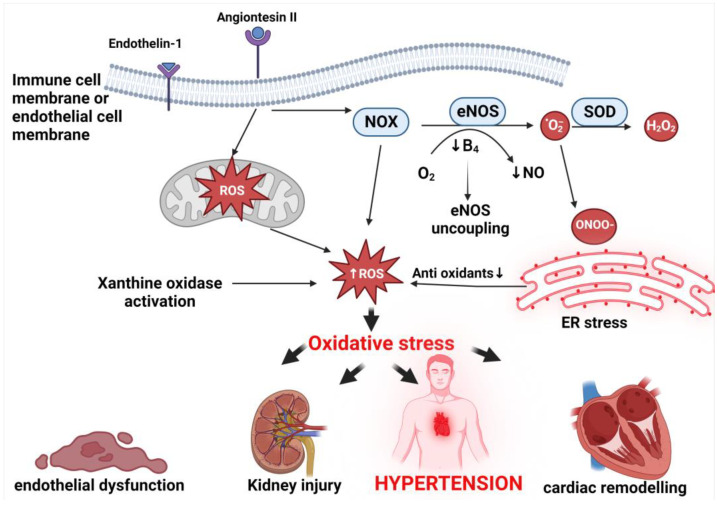
Proposed mechanisms of oxidative stress-induced hypertension. The NOX (NADPH oxidase) family and xanthine oxidase enzymes are the principal generators of reactive oxygen species (ROS) in hypertension, and they are regulated by pro-hypertensive and pro-inflammatory factors such as Ang II (angiotensin II) and ET-1 (endothelin-1). eNOS (endothelial nitric oxide synthase) uncoupling and mitochondrial and endoplasmic reticulum (ER) pathways affected by NOX/ROS also result in the production of ROS. These may result in hypertension due to endothelial damage, renal injury, or cardiovascular dysfunction. SOD, superoxide dismutase; NO, nitric oxide; BH4, tetrahydrobiopterin.

In hypertension, the important sources of ROS include non-phagocytic NADPH oxidase (NOX) hyperactivation, nitric oxide synthase (NOS) uncoupling, xanthine oxidase, mitochondrial stress, and endoplasmic reticulum stress (Figure 5). NADPH oxidase is a major source of ROS in the vasculature and kidney, which plays an important role in NO depletion, vascular damage, and endothelial dysfunction [94,95,96]. NADPH oxidase-derived superoxide inactivates NO in the process that generates peroxynitrite, leading to impaired endothelium-dependent vasodilation and hypertension [96]. eNOS activation normally produces NO; however, oxidation or deficiency of tetrahydrobiopterin (BH4) and L-arginine are associated with increased eNOS-mediated superoxide production as well as the decreased formation of vasoprotective NO [96,97]. Peroxynitrite oxidizes and destabilizes eNOS to produce more superoxide, whereas BH4 is susceptible to oxidation and upper oxide oxidizes it, uncoupling eNOS, causing endoplasmic reticulum stress and mitochondrial oxidative stress and producing more ROS [94,95]. Xanthine oxidase is an important source of ROS in the vascular endothelium and is associated with increased arteriolar tone and end-organ injury in hypertensive patients [98].

### 6.1. Endoplasmic Reticulum

The endoplasmic reticulum (ER) synthesizes, modifies, and delivers proteins to their target sites [99]. In a quality-controlled process, only correctly folded proteins are exported to the Golgi apparatus, whereas poorly folded proteins are maintained in the ER to complete the process or be degraded [100,101]. The ER’s protein load and folding capacity are balanced under physiological settings. Increased protein synthesis, accumulation of misfolded proteins, or changes in the ER’s calcium or redox balance cause ER stress, resulting in the activation of the unfolded protein response (UPR) [94,102]. Inositol-requiring protein 1 (IRE1), activating transcription factor 6 (ATF6), and protein kinase RNA-like endoplasmic reticulum kinase (PERK) are three primary axes of the UPR that, in response to ER stress, signal to downstream molecules [103]. As a consequence of protein folding, ROS are created in the ER, and certain ER stress conditions can promote ROS production in the ER [104]. ER stress stimulates signaling molecules, initiating the UPR and activation of Nox4, and possibly Nox2 during the UPR, generating more ROS [94]. The UPR causes the expansion of the ER membranes, an increase in the translation of folding chaperones, an acceleration in the destruction of unfolded proteins, and a decrease in the transcription and translation of the majority of other proteins, leading to apoptosis, phenotypic switching, de-differentiation, and trans-differentiation, all of which are mechanisms involved in cardiovascular remodeling and vascular damage in hypertension [103,105].

### 6.2. Mitochondrial Oxidative Stress

Mitochondria are responsible for the production of most cell adenosine triphosphate (ATP) through the electron transport chain’s enzyme complexes [33]. Electron transfer from one complex to the next is efficient and with minimal electron leakage, but in various disease conditions, electron leakage increases and can lead to a reduction of oxygen and the generation of superoxide and hydrogen peroxide [106,107]. In hypertension, mitochondrial dysfunction produces ROS, leading to oxidative stress [9]. The activation of angiotensin II (Ang II) stimulates the synthesis of mitochondrial ROS (mtROS) and the opening of the mitochondrial permeability transition pore (mPTP), which allows mtROS to leak into the cytosol [108]. In the cytosol, mtROS stimulates NOX via activating p38 MAPK and the JNK pathway or cSrc-dependent phosphorylation of p47phox [109]. NOX-derived ROS crosses the mitochondria, causing mitochondrial damage and the generation of mtROS, resulting in mtROS accumulation that leads to immune cell infiltration, Ang II-mediated eNOS uncoupling, reduced circulatory NO, and endothelial dysfunction, all of which cause adverse cardiovascular effects [94,108].

### 6.3. Nitric Oxide Synthase Uncoupling

Nitric oxide mediates vascular effects, and its synthesis requires l-arginine (substrate), while the co-substrates that are required are molecular oxygen, reduced NADPH, and the cofactor BH4 to stabilize eNOS [110,111]. Under conditions of oxidative stress, NOS removes an electron from NADPH and donates it to O_2_, which results in the production of O_2_– rather than NO. [94,112]. The uncoupling of eNOS, which is caused by a lack of BH4, has been linked to several cardiovascular diseases, including hypertension and aortic aneurysms [113,114]. Because BH4 is the regulator of all NOS isoforms, any of them can “uncouple” when subjected to stressful conditions. Tryptophan 447, which is located in the BH4-binding domain of eNOS, is an important component of the equation that determines whether eNOS generates NO or O_2_–. When this is mutated, the connection between BH4 and eNOS is disrupted, which results in the preferential formation of O_2_– [94]. Oxidative stress is the most important factor promoting NOS uncoupling, resulting in reduced NO production and increased O_2_– [95]. Oxidative stress has been demonstrated in spontaneous (genetic) and experimental models of hypertension, with increased p22phox mRNA expression and NADH/NADPH oxidase activity in the aortic and mesenteric vessels of stroke-prone spontaneously hypertensive rats [115]. Vascular oxidative stress has also been demonstrated in many forms of experimentally induced hypertension, such as Ang II-mediated hypertension, Dahl salt-sensitive hypertension, lead-induced hypertension, obesity-associated hypertension, aldosterone-provoked hypertension, and nitric oxide synthase inhibitor-induced hypertension [94,106].

Oxidative damage to the endothelium affects the circulation level of NO due to a decline in synthesis caused by the uncoupling of eNOS and the depletion of BH4. Increased production of ONOO– through NO–O_2_ coupling also adds to NO depletion [97]. ROS-induced reduction in circulatory NO due to endothelial dysfunction impairs the formation of the capillary network and blood flow regulation, resulting in decreased microcirculation in metabolically active tissues as well as dysregulations of glucose and dyslipidemia [116].

In prediabetic individuals, increased glucose levels are responsible for the activation of oxidative stress, which in turn leads to insulin resistance. Obesity has shown a substantial relationship with insulin resistance. In this context, an adipocyte-α derived factor, such as TNF-, leptin, FFA, and resistin, could be the mediator of oxidative stress-induced insulin resistance in the pre-diabetic condition [117,118]. In obesity, the formation of reactive oxygen species is increased and lipid peroxidation is induced in the adipocytes, liver, and skeletal muscles [56,119]. Increased FFA concentrations result in mitochondrial malfunction, including uncouplers of oxidative phosphorylation in mitochondria and increased superoxide formation, creating oxidative stress and decreasing intracellular glutathione to compromise natural antioxidant defenses [119].

## 7. Mechanisms of Oxidative Stress Associated with Impaired Fasting Glucose and Insulin Resistance

Insulin is secreted by the pancreas and drives nutrient transport into cells, acutely affects metabolic enzyme activity, regulates metabolic gene transcription, controls cellular development and differentiation, and regulates its clearance by activating receptors [120,121]. Oxidative stress contributes to numerous chronic conditions, including insulin resistance and type 2 diabetes [122]. Insulin resistance is common worldwide and can most accurately predict the development of diabetes [123]. In this situation, there is a decrease in peripheral insulin sensitivity [122]. An accumulation of oxidants is linked to the multifactorial etiology of insulin resistance, mainly in skeletal muscle and adipose tissue. ROS production mechanisms are many, including oxidative phosphorylation, transition metal ions, oxidase activity, protein folding, thymidine, and polyamine catabolism [124]. However, mitochondrial H_2_O_2_ production and NADPH oxidase activation are relevant to insulin resistance [125]. The mitochondrion is one cellular location that has a high capacity for the synthesis of oxidants such as H_2_O_2_ and other reactive oxygen species [126]. ROS and RNS have been found to disrupt the insulin signaling cascade; however, the disruption depends on the dose and is time-dependent [127]. When insulin is released, a burst of H_2_O_2_ is made, which exposes cells to ROS for a short time and at a low dose. This improves the insulin cascade by reducing the activity of tyrosine phosphatase, which raises the basal level of tyrosine phosphorylation in both the insulin receptor and the proteins it controls [20]. Studies have shown that oxidative stress impairs insulin signaling and leads to insulin resistance [20,128]. The proposed mechanisms leading to insulin resistance include the accumulation of specific lipid mediators, abnormal features of mitochondrial function, an increase in stress-activated protein c-Jun-N-terminal-kinase (JNK), and inflammatory pathways.

### 7.1. Lipid-Induced Insulin Resistance

Diacylglycerols (DAG) and ceramides mediate liver and skeletal muscle lipid-induced insulin resistance [20]. In insulin-resistant individuals, lipid oversupply from high-fat, high-calorie meals or excessive adipose lipolysis can contribute to enhanced fatty acid oxidation and worsening insulin resistance [129,130]. In contrast, a reduction of circulating FA levels with the lipolysis inhibitor acipimox increases insulin sensitivity, which correlates with a decrease in intramyocellular FA CoA concentration [131].

In individuals with prolonged increased triglycerides, DAG accumulates and impairs insulin signaling by activating conventional (α, βI, βII, γ) and protein kinase C (PKC) isoforms (δ, ε, v, θ) [20,131]. For increased intrahepatic triglyceride (IHTG), activation of the ε isoform (PKCε) is most consistently observed, and skeletal muscle PKCβ is observed [20,131]. PKC phosphorylates Thr1160 on the insulin receptor (INSR), destabilizing the insulin receptor kinase’s (IRK) active conformation and function and resulting in a defect in glucose transport or phosphorylation [20,131]. Lipid peroxidation is another mechanism that exacerbates insulin resistance. The two most prevalent ROS that are known to affect lipids are hydroxyl radicals and hydroperoxyl radicals [122]. Cells produce approximately 50 hydroxyl radicals in a second, and in a full day, each cell generates around 4 million hydroxyl radicals. These produced radicals have a detrimental effect on the biomolecules [122]. These radicals are known to cause unspecific damage to a biomolecule that is present a few nanometers from the site of their synthesis and lead to adjacent organelle damage and also plasma membrane damage, which is a key target in the signaling of tyrosine kinase and downstream effects signal transduction of many reactions, including insulin receptor substrate 1 (IRS-1), which is responsible for phosphorylation of another enzyme, PI3-kinase [132,133]. High levels of HO_2_ can also precipitate continuous peroxidation because this molecule alone has a strong oxidant effect and could initiate the chain reaction of oxidation of polyunsaturated phospholipids, hence impairing membrane function [133]. Damage to the plasma membrane leads to an inability of the glucose transporter to function as well as the entire mechanism of phosphorylation, thereby affecting insulin function [122].

### 7.2. Mitochondrial Dysfunction

Mitochondria control glucose sensing and insulin secretion in beta cells [122]. Mitochondrial dysfunction has been recognized to cause insulin resistance and is an underlying cause of diabetes [134]. Insulin secretion by pancreatic beta cells is linked to the extracellular glucose concentration, which is phosphorylated by glucokinase and metabolized to pyruvate in mitochondria [133]. Pyruvate enters mitochondria and is oxidized by tricarboxylic acid (TCA) to NADH and FADH2, which donate electrons to the electron transport chain, leading to ATP generation. Mitochondrial ATP is transported to the cytosol, raising the cytosolic ATP/ADP ratio, leading to depolarization and exocytosis of insulin-containing vesicles [135,136]. Mitochondrial dysfunctions impair this metabolic process and promote apoptosis and beta-cell death. Many human studies have shown that mitochondrial dysfunction exists in obese and insulin-resistant patients, with these individuals having downregulated metabolic and mitochondrial pathways in obesity and insulin resistance [137,138]. ROS generation in beta cells is proposed to be caused by hyperglycemia, hyperlipidemia, hypoxia, and ER stress [139]. Mitochondria can contribute to fatty acid inflow and the activation of stress-related kinases, both of which can lead to insulin resistance [132,140]. Oxidative stress appears to have a significant role in mitochondrial malfunction, which can amplify stress signals and limit adenosine triphosphate (ATP) synthesis [137].

Insulin release from beta cells is triggered by mitochondrial oxidative phosphorylation (OxPhos) and ATP production [141]. Beta cells from patients with T2DM showed decreased OxPhos gene expression [135,139]. In T2DM, IR and chronic hyperglycemia lead to increased glucose and fatty acid metabolism in beta cells [142]. Increased fatty acid levels and hyperglycemia increase NADH and FADH, which lead to the activity of the electron transport chain and ROS production, consequently leading to beta cell oxidative stress [141]. Increased fatty acids also cause incomplete fatty acid oxidation, which worsens ROS generation [143]. Oxidative stress predisposes mitochondrial damage and enhanced mitochondrial fission, leading to a further decline in OxPhos and increased ROS generation, leading to apoptosis and beta cell loss [139,144]. Beta cells are vulnerable to oxidative stress due to high ROS production and low antioxidative enzyme expression [139,145]. Human islets from diabetic individuals show that lipid peroxide protein adducts and lipid infusion increases islet ROS and impairs insulin secretion, leading to mitochondrial dysfunction [122,146].

### 7.3. Low-Grade Inflammation

The production of excess ROS leads to oxidative stress and activates numerous transcription factors, including NF-κB, JNK/SAPK, and MAPK [147]. The NF-κB transcription factor plays a role in mediating immune and inflammatory responses by elevating systemic pro-inflammatory cytokines and promoting an insulin-resistant environment through the activation of activated protein kinase C (PKC) [56,147]. The NF-B pathway is triggered by an active serine kinase, IKK, phosphorylating the inhibitory subunit, IkB [148,149]. When exposed to an oxidative environment, mitogen-activated protein kinases (MAPK), such as JNK, ERK, and p38 MAPK, are activated [131,150]. Increased serine-threonine phosphorylation impairs the protein’s ability to recruit and activate downstream SH-2-containing signaling molecules and disrupts the insulin receptor substrate (IRS) protein’s ability to interact with the insulin receptor, according to the proposed mechanism of insulin signal interference by activated serine/threonine kinases [151,152].

### 7.4. Glucose Transporters

The diffusion of glucose into the cell is facilitated by glucose transport (GLUT), and GLUT4 is the principal glucose transporter in adipose tissue, skeletal muscle, and cardiac muscle [153]. The insulin binds to insulin receptors and activates a signal transduction cascade that leads to enhanced GLUT4 expression in the plasma membrane, hence enhancing glucose uptake from the circulation [122,153,154]. An increased metabolite flow into mitochondria, changes in mitochondrial proteins, and decreased expression of antioxidant enzymes can lead to higher ROS levels in obese and diabetic conditions [155]. ROS causes insulin resistance in the periphery by impairing insulin receptor signal transduction and decreasing cellular membrane GLUT4 transporter expression [125,155,156]. Normal glucose tolerance is sustained in the early stages by compensatory hyperinsulinemia, eventually leading to desensitization of the peripheral tissues to insulin [56]. This regulation employs distinct transduction proteins compared to the typical pathway. The signaling of protease inhibitor 3 (PI3)-kinase shifts above optimal insulin concentrations. Instead of phosphorylating phosphatidylinositol 4,5-bisphosphate (PIP2), PI3-kinase phosphorylates Rac, and hence raising NOX4 activity. NOX4 is a potent oxidizing enzyme that generates reactive oxygen species, increasing ROS [132]. Oxidative stress causes Casein kinase-2 (CK2) to activate the retromer, which, instead of the plasma membrane, the retromer signals the trans-Golgi network to transport GLUT4 to lysosomes for destruction, resulting in hyperglycemia [132,154].

## 8. Immune Activation Mechanisms of Oxidative Stress in Metabolic Syndrome

Chronic inflammation in metabolic syndrome is thought to be mainly mediated by adipose tissue, and involves a crosstalk between various cell components such as adipocytes, T cells, macrophages, dendritic cells, B cells, and fibroblasts [89,157]. Non-obese adipose tissue mainly contains type 2 macrophages, which express anti-inflammatory cytokines such as IL-10 and transforming growth factor-β [158]. In obesity, there is an increased infiltration of M1 macrophages derived from the bone marrow that particularly express pro-inflammatory cytokines [158]. Saturated fatty acids from adipocytes activate M1 macrophage toll-like receptor 4 and macrophage-inducible C-type lectin via an integrated stress response involving the activation of the NF-κB pathway [158]. This results in the secretion of inflammatory cytokines such as TNF-α, IL-6, and IL-1, which recruit further pro-inflammatory immune cells such as CD4+ and CD8+ T cells, natural killer (NK) cells, and innate lymphoid cells into the adipose tissue to boost the immune response [157,158,159]. These inflammatory cytokines mediate insulin resistance in metabolic syndrome, especially macrophage-derived IL1-β [160]. T cells also play an important role in inducing insulin resistance in adipose tissue. McDonnell et al. demonstrated that CD8+ T cells infiltrate the adipose tissue of obese mice, where they accumulate, are clonally expanded, and activate in response to isolevuglandin-containing M2 macrophages [161].

Furthermore, initial inflammatory cytokines are imperative in the propagation of chronic inflammation stimulated by oxidative stress and their intracellular status. ROS cause initial damage to the mitochondria, and this leads to the activation of the nod-like receptor family pyrin domain containing 3 (NLRP3), which is an inflammasome [162]. This inflammasome is a key molecule in the signaling of IL-1β expression by macrophages. Additionally, oxidative damage to DNA induces several molecules, including inflammatory molecules, involved in gene expression [162,163]. Expression of isoprostane due to peroxidation by ROS leads to further expression of interleukin-8 (IL-8), a chemoattractant cytokine that attracts several inflammatory cells, including neutrophils. Hence, continual expression of this molecule leads to a prolonged state of inflammation [153]. Although not clearly understood, other studies have indicated that ROS lead to the activation of an enzyme called peroxiredoxin-2 (PRDX2), which has an effect on macrophages, triggering them to produce and release TNF-α, a key cytokine in chronic inflammation [154].

The hallmark of oxidative stress associated with continued immune cell proliferation and activation in adipose tissue is the suppression of bioenergetics and a metabolic switch to preferential utilization of select catabolic pathways for their energy needs [164]. More importantly, owing to the increased dynamic bioenergetic demands of activated cells in the adipose tissue of obese individuals, there is a concomitant increase in mitochondrial activity to produce ATP, a situation that increases ROS production and contributes to chronic inflammation in ways already described above [164]. Thus, immune activation contributes to metabolic syndrome via inflammatory cytokines that induce insulin resistance as well as the generation of ROS that promote apoptosis, inflammation and metabolic dysfunction [9,27,28]. However, the exact underlying mechanisms involving immunometabolism and ROS signaling remain unclear to date.

## 9. Gut Microbiota, Oxidative Stress, and Metabolic Syndrome

**Figure 6:**
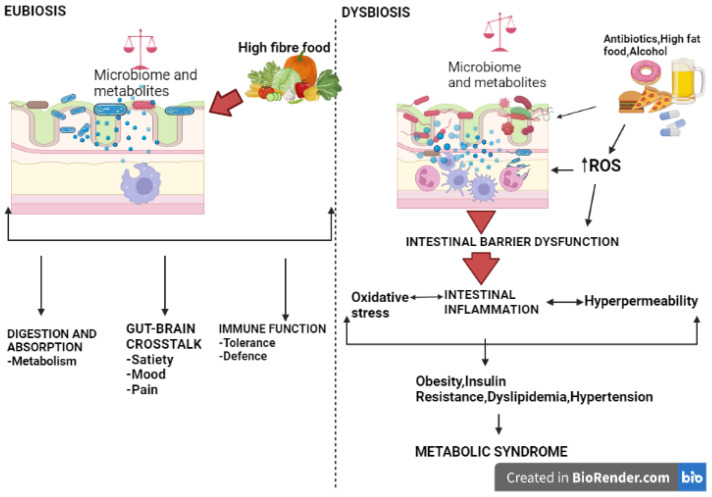
Eubiosis and dysbiosis in the gut. In normal conditions, the gut is in a eubiotic state, having a pool of microbes that is mostly composed of non-pathogenic microorganisms that are relevant for normal physiological function, such as promoting physiological cross-talk with other systems such as the brain, cardiovascular organs, and metabolic-related tissues, helping to avoid and fight hypertension and metabolic syndrome progression. The gut microbiota produces compounds beneficial to host intestinal health, which can be regulated through personal nutrition. However, dysbiosis in the gut microbiota (triggered and caused by antibiotics, urban diet, and sedentary lifestyle) is linked to chronic inflammation and exacerbates oxidative stress, consequently leading to metabolic syndrome.

The gut microbiota is the most diverse microbial community in the human body, with more than 1000 species encoding approximately 3 million genes [165]. The gut microbiota interacts with the host’s brain and targets organs through the autonomic nervous system and circulatory and endocrine systems [166]. The gut microbiota plays a key role in maintaining physiological function as it modulates host nutrition, energy harvest, epithelial homeostasis, the immune system, and drug metabolism while maintaining balance [167]. Dysbiosis, an imbalance of gut microbiota content resulting in increased pathological species, can be caused by infections, antibiotic therapy, diseases, diet, and lifestyle (Figure 6) [150,168,169]. Dysbiosis of the gut microbiota increases the risk of metabolic syndrome by causing inflammation, increasing reactive oxygen species, and oxidative stress [170,171]. Intestinal dysbiosis causes intestinal permeability, which can lead to metabolic endotoxemia, which is a cause of chronic low-grade systemic inflammation [171,172]. Modulation of dysbiosis through dietary interventions and probiotic supplementation may help treat metabolic syndrome [173].

The symbiotic association between host-microbe interactions in the intestine determines the oxidative stress level, which is influenced by the balance between good and harmful gut microbiota [174,175]. Lactobacillus brevis 23017, Bacillus SCo6, Lactobacillus plantarum, and Macleaya cordata extract can reduce the production of oxidative stress and protect the intestinal mucosal barrier [176,177,178,179,180]. The composition of gut microbiota and gut cells is directly correlated with ROS production in the host body [181]. Under healthy conditions, there is a dynamic equilibrium between ROS formation and elimination from the host body, and ROS harbor microbicidal machinery in innate cells. An imbalance between the production of ROS and antioxidants can lead to oxidative stress, disrupting redox signals, and intestinal damage [182,183]. The gut microbiome has been associated with the pathophysiology of most chronic diseases, such as obesity, diabetes, dyslipidemia, and hypertension, which can consequently result in the metabolic syndrome [165,183,184,185].

Obesity increases the risk of chronic metabolic disorders, and there is evidence that the gut microbiota plays an important role in the development of obesity, including interactions between the gut microbiota and host metabolism [186]. Studies in both animals and humans have shown that the composition of the gut microbiota in healthy individuals is significantly different from that in individuals with the above-mentioned conditions, suggesting that the gut microbiota may play an important role in their development. Studies using 16S rRNA pyro-sequencing have shown that the composition of the gut microbiota of obese animals and humans differs from that of healthy, leaner individuals [187]. Obesity has been associated with two dominant bacterial phyla, *Firmicutes* and *Bacteroidetes*, with the *Firmicutes*/*Bacteroidetes* ratio increasing significantly in obese mice and humans [188].

In studies focusing on diabetes mellitus, evidence suggests that microbiota can affect glucose metabolism in both preclinical and healthy animals. Genera of *Bifidobacterium*, *Bacteroides*, *Faecalibacterium*, *Akkermansia*, and *Roseburia* were negatively associated with T2DM, while *Ruminococcus*, *Fusobacterium*, and *Blautia* were positively associated [186]. Experimental studies in animals and humans found that a high-calorie diet is a causal factor in obesity and may induce changes in the function of the gut microbiome [188]. These studies have shown that the gut microbiota regulates fat accumulation in the host, which influences obesity [185,189,190,191].

## 10. Comorbidities Associated with Risk for Metabolic Syndrome

Having metabolic syndrome can increase the risk of developing T2DM, CVD, diabetes, polycystic ovary syndrome (PCOS), nonalcoholic fatty liver disease (NAFLD), chronic kidney disease, some types of cancer (breast, uterus, colon, esophageal, pancreatic, kidney, and prostate cancers), and osteoarthritis [18]. Many conditions are implicated in the development of metabolic syndrome and are known to coexist with each other in its development [192]. Hence, screening for comorbidity should be an integral part of metabolic syndrome care, as further studies confirm the association and the underlying mechanisms of metabolic syndrome and its comorbidities [193,194].

### Metabolic Syndrome and Cardiovascular Disease Risk

A spectrum of cardiovascular conditions, such as microvascular dysfunction, coronary atherosclerosis and calcification, cardiac dysfunction, myocardial infarction, and heart failure, are all related to metabolic syndrome [195]. Each component of the metabolic syndrome is a separate risk factor for cardiovascular disease, and the combination of these risk factors increases the rates and severity of cardiovascular disease [195]. For instance, a study by Klein et al. reported that patients with a single metabolic syndrome component had a 2.5% risk of developing CVD in 5-years, while patients with ≥4 components had about a 14.9% risk of developing CVDs [18].

Compared to other risk factors of metabolic syndrome, hypertension is not only considered a major risk factor of CVD; it is regarded as a key feature of metabolic syndrome and is also attributed to about one-third of all deaths worldwide [196]. An increase in hypertension amplifies the effect of cardiovascular cellular damage and can eventually compromise the performance of the kidneys and lungs, which are key organs in the development of CVD and eventually metabolic syndrome [1,197]. Studies have shown that an amplified effect of metabolic syndrome is set into motion as a result of an overreaction due to overstimulation of the sympathetic nervous system (SNS) [193]. The overreaction of the SNS results in the stimulation of the renin-angiotensin-aldosterone system (RAAS), alterations in adipose-derived cytokines such as leptin, insulin resistance, and structural as well as functional renal changes [194]. These will ultimately collectively amplify the activity of both the physiologic functions of the SNS, which will eventually increase blood pressure [194,198]. Additionally, the RAAS also indirectly raises blood pressure by acting on the water retention system, thereby causing a surge in blood pressure which is an independent and important risk factor of metabolic syndrome development [194]. Triglycerides alone are an independent factor that contributes to many conditions that are directly and indirectly associated with metabolic syndrome and CVD. Triglycerides are a risk factor for CVD events, independent of serum HDL or low-density lipoprotein (LDL) levels [199]. Triglycerides increase the likelihood of obesity, which is a direct predisposing factor to metabolic syndrome. Therefore, triglycerides are directly associated with the development of diabetes, obesity, atherosclerotic cardiovascular disease, and hence metabolic syndrome [199,200].

## 11. Biomarkers of Oxidative Stress in Metabolic Syndrome

Oxidative stress biomarkers include molecules altered by ROS in the microenvironment and antioxidant system molecules that alter with redox stress [201]. Risk factors disrupt cell signaling pathways, increasing inflammatory markers, lipid peroxides, and free radicals, producing cell damage and clinical signs of metabolic syndrome. It is hypothesized that oxidative stress and inflammatory markers contribute to metabolic syndrome pathogenesis [6,14]. Quantification of biomarkers is the most accurate way to determine the amount of oxidative stress present in vivo. Total antioxidant capacity can also be used as a measure of oxidative stress in metabolic syndrome [14,202]. The isoprostanes (IsoP) generated from arachidonic acid, specifically 8-iso prostaglandin-F2alpha (8-isoPGF2), could be a good measure for investigating simultaneously oxidative stress and inflammation in disorders in which both are thought to be implicated [14,203]. Various studies have been conducted in individuals with metabolic syndrome in which the concentrations of oxidative stress biomarkers and antioxidant enzyme activity were measured simultaneously. The findings reveal that the presence of metabolic syndrome is related to an increase in oxidative stress biomarkers and a decrease in antioxidant capacity, which shows that metabolic syndrome is linked to a pro-inflammatory state and poor health as part of a very complex process driving cardiometabolic diseases [14,203,204,205,206,207].

Biomarkers of oxidative stress are utilized in studies to determine patients at risk of complications and administration of the right therapy to reduce the burden of metabolic syndrome. The markers of oxidative stress include biomarkers of lipid peroxidation, protein and amino acid oxidation, and DNA oxidation [201]. Thiobarbituric acid-reactive substances (TBARS), malondyadehide (MDA), 4-hydroxy-2-nonenal (4-HNE), and F2-isoproteines are markers used to determine the presence of lipid peroxidation, which is an indicator of oxidative stress. Protein carbonyls, advanced glycation (AGEs), oxidized LDL (ox-LDL), and advanced oxidation proteins indicate protein oxidation. DNA oxidation markers include 8-oxo-2 deoxyguanosine (8-0xo-Dg),5-chlorouracil, and 5-chlorocytosine [201,206,208,209].

The markers associated with ROS generation are xanthine oxidase, gamma-glutamyl transferase (GGT), myeloperoxidase (MPO), NOX, and NOS [202]. Gamma-glutamyl transferase (GGT) is an enzyme found in many parts of the body, such as the kidney, pancreas, liver, spleen, heart, and brain. It recycles precursors to glutathione (GSH), which is an antioxidant and metabolic substrate. Metabolic syndrome, diabetes, high blood pressure, and stroke risk can all be predicted by a raised GGT [13,210]. The non-enzymatic markers include glycoprotein A (GPA), C-reactive protein (CRP), ferritin, and uric acid. CRP is a non-specific biomarker used to assess disease activity, diagnose, and classify inflammatory disorders such as rheumatic diseases [211]. Dyslipidemia, diabetes, and metabolic syndrome are linked to elevated CRP [211]. Individuals with metabolic syndrome have high serum ferritin without transferrin saturation [212]. Serum ferritin correlated positively with two indicators of oxidative stress: liver damage and insulin resistance [212]. Here, serum ferritin levels are important in metabolic syndrome diagnosis [212].

Other useful biomarkers that are significantly positively associated with metabolic syndrome include adipokines such as adiponectin and lectins. Adipose tissue expresses adiponectin levels, and levels are inversely related to the degree of adiposity [213,214,215]. Adiponectin is a well-known and accepted marker for metabolic syndrome and diabetes [213,214,215]. Decreased levels of adiponectin in the serum have been linked to the development of metabolic syndrome, and others have suggested its use to predict metabolic syndrome [213,214,215]. There is a connection between the hormone leptin, insulin resistance, and abdominal obesity. Leptin is a hormone that regulates energy metabolism. According to many studies, a substantial positive association exists between levels of leptin and metabolic syndrome. The presence of high leptin levels has been proposed as a potential marker for the development of metabolic syndrome [216,217].

## 12. Targeted Therapeutic Strategies for Metabolic Disease

Most of the biomarkers used in the detection of metabolic syndrome are not specific. This is because most of the products of oxidative stress are unstable and have a short half-life in the bloodstream [160]. Although most of the biomarkers are not specific, studies have shown that indirect methods for the detection of metabolic syndrome are reliable and depend on certain macromolecules such as DNA, lipids, and proteins, as these molecules experience significant damage due to oxidative stress [218]. Multidisciplinary strategies are needed to prevent and manage metabolic diseases, including lifestyle interventions and surgical or pharmacotherapeutic approaches [219].

Nutrition is a major environmental factor contributing to metabolic syndrome [220]. Westernization of lifestyles has led to an increase in convenience foods, fast-food availability, food marketing, and larger food portions, leading to metabolic syndrome worldwide [219]. Different therapeutic strategies have been suggested to counter the effects of reactive oxygen species. However, only a few strategies have been elaborated, most of which utilize macromolecules at different levels of biosynthetic pathways [221]. Several studies have shown that the type of diet influences the gut microbiota. An example is the Western diet, which decreases microbial richness and increases the *Firmicutes*/*Bacteroidetes* ratio, while a diet rich in -3 polyunsaturated fatty acids (PUFAs) is associated with anti-inflammatory effects [171]. Several studies have found that replacing energy intake from saturated fatty acid with equivalent energy intake from Polyunsaturated fat (PUFA) and monounsaturated fat (MUFA) or high-quality carbohydrate such as whole grains can lower CVD risk [222]. MUFA can inhibit adipose (NLRP3) inflammasome-mediated IL-1β secretion, NLRP3 secretion, and insulin resistance, even in mice with diet-induced obesity [223]. Fruits, vegetables, legumes, and whole grains are appropriate sources for cardioprotective components [2]. The Mediterranean diet, consisting of fruits, vegetables, olive oil, red wine, nuts, and other food components, has been reported to have beneficial effects on longevity and ameliorating metabolic syndrome [224]. The Mediterranean diet contains natural antioxidants and bioactive compounds such as polyphenols such as naringenin, apigenin, and ellagic acid from olives, which have beneficial properties that lower the risk for metabolic syndrome and CVD [225,226]. One of the mechanisms of action involved in reducing the risk for the development of metabolic syndrome by polyphenols is inhibiting the inflammasome and NF-кB, leading to a decrease in the secretion of proinflammatory cytokines [227]. Nuts, such as almonds and walnuts, reduce inflammation and oxidative stress by decreasing the levels of C-reactive protein, IL-6, endothelial adhesion molecules, and oxLDL, thereby reducing the risk for metabolic syndrome [228,229,230]. Tocopherols, key lipophilic radical-scavenging antioxidants, could interrupt the lipid peroxidation cycle and modulate the nuclear factor erythroid 2/electrophile-responsive element (Nrf2/EpRE), PI3K/Akt/mTOR, and NF-κB signaling pathways, and hence improving the quality of life [231].

Studies involving pharmacotherapeutic agents suggest that inhibition of protein synthesis, which has been activated as a result of reactive oxygen species, is one way that targeted therapy could be achieved, as elaborated by Vassalle et al. [232]. Most drugs with antioxidant properties, such as those used in the treatment of CVD, have simultaneous effects in relation to oxidative stress, i.e., beta blocks, angiotensin-converting enzymes (ACE), and angiotensin receptor blockers (ARB) have multiple effects on different pathways [233]. Therapeutic agents such as statins show both antioxidant and anti-inflammatory properties, as there is a reduction in cytokine production once administered [234]. Recent studies have shown that statins improve both blood vessel and heart-related diseases and achieve this by inhibiting specific proteins such as Rac and Rho [233,235]. The pharmacological effect of statins is through targeting and blocking a coenzyme known as hydroxylmethylgrutaryl A reductase. This in turn will lead to the inhibition of the biosynthesis of mevalonic acid, which is a progeny of nonsteroidal isoprenoids and a lipid molecule where Rac and Rho attach [236]. Furthermore, Rho acts on the endothelium and negatively moderates nitric oxide synthase, while on the other hand, Rac acts as a key target for the organization and action of NADPH oxidase [234,236]. NADPH oxidase is a precursor of most reactive oxygen species. Statin has thus been known to target NADPH oxidase and negatively inhibit it, thereby reducing the production of ROS [234].

## 13. Conclusions

The mechanisms underlying metabolic syndrome mediated by oxidative stress are complex and intricately interrelated. Biomarkers of metabolic syndrome that explain disease severity are available. However, more clinical studies are required to understand their value and usage in the clinical setup.

## 14. What Is Known

- Oxidative stress plays a role in metabolic derangements in obesity, diabetes, and cardiovascular pathogenesis;
- Biomarkers and molecular targets may help us develop innovative methods for preventing, diagnosing, and treating inflammatory and metabolic disorders;
- Antioxidants can be used as a preventative or therapeutic treatment for metabolic diseases.

## 15. What Is New

- Mitochondrial oxidative stress and dysfunction may be the primary causes of oxidative damage and metabolic abnormalities in metabolic syndrome;
- Several signaling pathways involving NF-kB, PKC, MAPK, polyol, JNK, ERK, and NOX are activated to induce metabolic syndrome and multiple organ damage;
- Adiposity plays a vital role in inducing oxidative stress that results in endothelial dysfunction, cardiovascular remodeling, and hypertension;
- Components of the Mediterranean diet, such as polyphenols found in olives, can lower oxidative stress and reduce the risk of the development of metabolic syndrome.

## Data Availability

All data is contained within the manuscript.
